# Acute Physiology and Neurologic Outcomes after Brain Injury in SCOP/PHLPP1 KO Mice

**DOI:** 10.1038/s41598-018-25371-2

**Published:** 2018-05-08

**Authors:** Travis C. Jackson, C. Edward Dixon, Keri Janesko-Feldman, Vincent Vagni, Shawn E. Kotermanski, Edwin K. Jackson, Patrick M. Kochanek

**Affiliations:** 1 University of Pittsburgh School of Medicine, Safar Center for Resuscitation Research, Children’s Hospital of Pittsburgh of UPMC, John G. Rangos Research Center – 6th Floor 4401 Penn Avenue, Pittsburgh, PA 15224 USA; 2 University of Pittsburgh School of Medicine, Department of Critical Care Medicine, Scaife Hall, 3550 Terrace Street, Pittsburgh, USA; 3 University of Pittsburgh School of Medicine, Department of Neurology, 811 Kaufmann Medical Building, 3471 Fifth Avenue, Pittsburgh, USA; 4 University of Pittsburgh School of Medicine, Department of Pharmacology and Chemical Biology, Bridgeside Point Building 1, 100 Technology Drive, Pittsburgh, USA

## Abstract

Suprachiasmatic nucleus circadian oscillatory protein (SCOP) (a.k.a. PHLPP1) regulates long-term memory consolidation in the brain. Using a mouse model of controlled cortical impact (CCI) we tested if (1) brain tissue levels of SCOP/PHLPP1 increase after a traumatic brain injury (TBI), and (2) if SCOP/PHLPP1 gene knockout (KO) mice have improved (or worse) neurologic outcomes. Blood chemistry (pH, pCO_2_, pO_2_, pSO_2_, base excess, sodium bicarbonate, and osmolarity) and arterial pressure (MAP) differed in isoflurane anesthetized WT vs. KOs at baseline and up to 1 h post-injury. CCI injury increased cortical/hippocampal SCOP/PHLPP1 levels in WTs 7d and 14d post-injury. Injured KOs had higher brain tissue levels of phosphorylated AKT (pAKT) in cortex (14d post-injury), and higher levels of phosphorylated MEK (pMEK) in hippocampus (7d and 14d post-injury) and in cortex (7d post-injury). Consistent with an important role of SCOP/PHLPP1 on memory function, injured-KOs had near normal performance on the probe trial of the Morris water maze, whereas injured-WTs were impaired. CA1/CA3 hippocampal survival was lower in KOs vs. WTs 24 h post-injury but equivalent by 7d. No difference in 21d cortical lesion volume was detected. SCOP/PHLPP1 overexpression in cultured rat cortical neurons had no effect on 24 h cell death after a mechanical stretch-injury.

## Introduction

Traumatic brain injury (TBI) is a leading cause of death and disability in the young^1^. New therapies are desperately needed to improve neurologic outcomes in surviving patients with moderate-severe TBI^2^. An ideal therapy (1) limits acute secondary injury and (2) ameliorates chronic behavioral deficits. In this study we tested if gene deletion of suprachiasmatic nucleus circadian oscillatory protein (SCOP) (also termed PHLPP1) in mice improves recovery following a controlled cortical impact (CCI) injury.

Neuronal SCOP/PHLPP1 blocks membrane localized K-Ras (a small GTPase), which in turn inhibits the downstream kinase, mitogen activated protein kinase (MEK)^3^. MEK is a vital modulator of memory function in the brain; bilateral injection of the MEK inhibitor U0126 into the medial prefrontal cortex impairs memory consolidation as well as long-term retrieval on the MWM task but does not affect acquisition (i.e. learning)^4^. Thus, MEK activation in rodents links the cell signaling pathways involved in hippocampal spatial memory processing with consolidation of the engram in the frontal cortex.

Disrupting SCOP/PHLPP1 signaling in the brain alters MEK activation and impairs memory formation. Transgenic mice overexpressing SCOP/PHLPP1 in the forebrain had impaired long-term memory consolidation on a novel object recognition task (i.e. impaired recall to identify the novel object 24 h post-training) but normal acquisition (i.e. overexpression did not affect learning)^5^. Furthermore, in naïve WT mice, training on novel object recognition resulted in endogenous calpain-mediated degradation of hippocampal SCOP/PHLPP1 and increased MEK activation^5^. Thus, initial studies suggested that therapeutic inhibition of SCOP/PHLPP1 might be a useful strategy to enhance memory functions. However, it was later reported that conditional KO (gene deletion) of SCOP/PHLPP1 in the mouse neocortex/hippocampus also impaired long-term memory on a novel object recognition task^6^. Finally, crossbreeding whole body SCOP/PHLPP1 KOs with calpain 1 KOs, reversed memory deficits normally seen in the latter on a fear-conditioning paradigm^7^. Thus, SCOP/PHLPP1 levels that are too high or low lead to behavioral impairments. To the best of our knowledge it has not been tested if (1) SCOP/PHLPP1 levels are disturbed after a TBI, (2) if memory deficits induced by acute brain injury are improved or exacerbated in SCOP/PHLPP1 KOs, and (3) if naïve KOs have normal or altered memory function as assessed by the MWM learning and memory paradigm.

Protein kinase B (AKT) survival signaling is a second major target of SCOP/PHLPP1 in neurons. SCOP/PHLPP1 inhibits AKT which promotes apoptotic cell death^8,9^. *In vitro* studies in primary cortical neurons show that normal synaptic activity leads to calpain mediated SCOP/PHLPP1 degradation, which in turn activates AKT, and protects against subsequent apoptotic mechanisms induced by starvation or oxidative stress^9^. Furthermore, cerebral infarct volume in a model of stroke is decreased in whole body SCOP/PHLPP1 KOs 24 h post-injury vs. WTs, and neuroprotection is reversed by co-administration of an AKT inhibitor^10^. Thus, SCOP/PHLPP1 KOs may also have improved histological outcomes after a TBI; however, the evolution of secondary cell death pathways after a TBI vs. stroke differs, which could influence the therapeutic efficacy of AKT activation in the setting of trauma^11^.

In this study we report several novel findings: (1) we identified differences in baseline and post-injury physiology in isoflurane anesthetized KOs vs. WTs, (2) baseline MWM performance is similar in naïve KOs vs. WTs, (3) after a CCI, hippocampal/cortical pMEK is increased in injured KOs vs. WTs and is associated with improved performance on the probe trial (memory component) of the MWM, and (4) after a CCI, long-term histological outcomes are equivalent in KOs vs. WTs. Our findings add to a growing body of evidence which supports SCOP/PHLPP1 targeting as a novel therapeutic strategy to improve neurological recovery after brain injury.

## Results

### SCOP/PHLPP1 KO vs WT Physiology

Acute physiology after a CCI was assessed using our standard protocol^12–14^. Baseline physiology has not been reported for any of the three currently available SCOP/PHLPP1 transgenic mice^5,6,10^. Also, whole body SCOP/PHLPP1 KOs are the most widely studied of the various strains and have been investigated in the setting of cancer, immunity, cardiovascular disease, and in stroke^7,10,15–18^.

**Figure 1:**
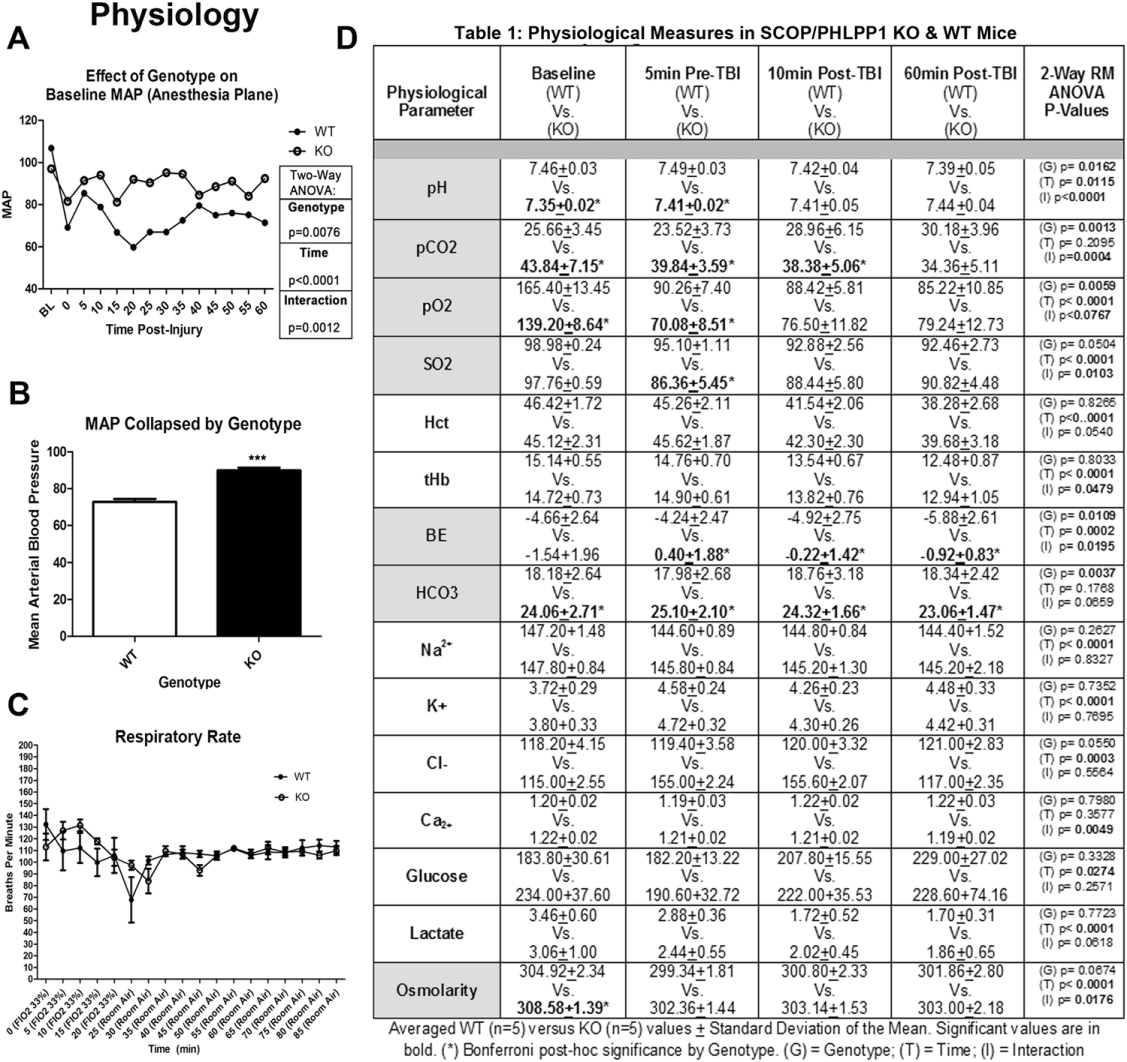
Differences in Acute Physiology of SCOP/PHLPP1 KO Mice. (**A**) MAP (mmHG) in anesthetized WT vs. KO mice at baseline (BL) and up to 1 h after a CCI. (**B**) Shows the average MAP value collapsed across time by genotype. (**C**) Respiratory rate of WT vs. KO mice on an adjusted FIO2 of 33%, or in room air. (**D**) Blood gas and chemistry in anesthetized WT vs. KO mice. Measurements were taken at baseline (BL), after surgical manipulations in preparation for a CCI (pre-TBI), and 10–60 min after a CCI (Post-TBI). MAP and blood chemistry were analyzed by Two-way-ANOVA (repeated measures for MAP); MAP data collapsed by genotype was analyzed by unpaired t-test; (***)p < 0.0001. Data were significant at p < 0.05. Graphs show mean + SEM. White bars/closed circles indicate WT genotype. Dark bars/open circles indicate KO genotype. Highlighted (grey) cells within the table indicate blood chemistry/gas targets which differed by genotype.

A number of physiological differences were observed between KOs vs. WTs at baseline (prior to injury) under isoflurane/nitrous oxide anesthesia. Bicarbonate and PaCO_2_ was increased in KOs vs. WTs. Conversely, PaO_2_ was increased in WTs vs. KOs (Fig. 1D). Whole blood pH was slightly alkalotic in WTs vs. KOs. Finally, baseline osmolarity also mildly differed between WTs vs. KOs (Fig. 1D).

Additional (new) physiological differences presented after a CCI, whereas others observed at baseline resolved during the post-injury period. Pre-injury mean arterial pressure (MAP) did not differ between genotypes. Consistent with the effect of a CCI on arterial pressure in mice^13^, MAP immediately decreased following insult in WTs. Lower pressures were maintained in WTs up to 1 h post-injury (Fig. 1A). In contrast, the drop in MAP post-injury was blunted in KOs (Fig. 1A). This averaged to a ~17 mmHg increase in KOs vs. WTs over the 1 h post-injury period (Fig. 1B). Injured KOs continued to have higher PaCO_2_, sodium bicarbonate (HCO3), base excess (BE), and blood osmolarity vs. WTs after CCI (Fig. 1D). In contrast, by 10 min post-injury, differences in pH, PaO2, and osmolarity resolved and were equivalent in KOs vs. WTs (Fig. 1D). Respiratory rate did not differ by genotype (Fig. 1C).

### Brain SCOP/PHLPP1 levels increase after a TBI

**Figure 2:**
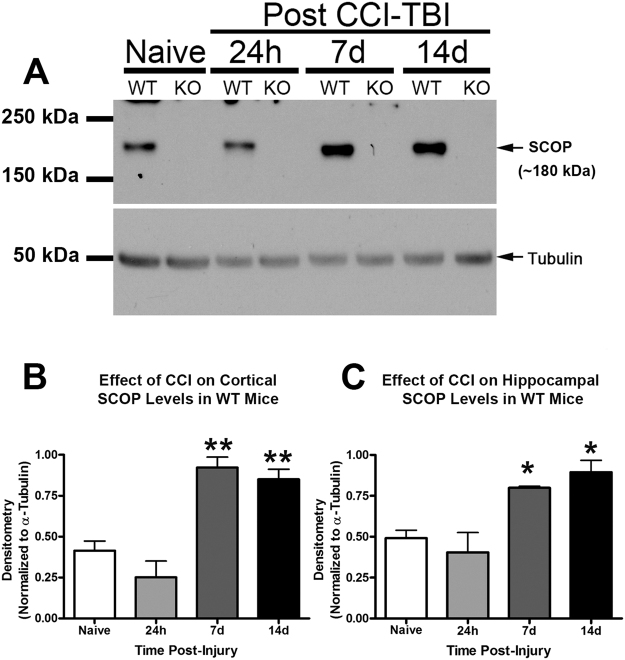
SCOP/PHLPP1 is increased in WT cortex and hippocampus after a TBI. Hippocampal and cortical tissue were harvested from WT/KO mice for protein analysis. (**A**) Representative Western blot shows increased SCOP/PHLPP1 protein in the ipsilateral (injured) hippocampus of WT mice at 7d and 14d post-CCI. Western blots also confirm absence of SCOP/PHLPP1 in the hippocampus of KO mice. Cropped blots are from the same gel (**B** and **C**) Semi-quantitative densitometry of SCOP/PHLPP1 levels in the cortex (n = 3/group; p = 0.0004) and hippocampus (n = 3/group; p = 0.0044) of WT mice at 7d and 14d post-injury. Data were analyzed by One-Way-ANOVA followed by the Newman-Keuls post hoc test. Data were significant at (*)p < 0.05 and (**)p < 0.001. Asterisks indicate significant difference vs. naïve mice. Graphs show mean + SEM.

We reported that SCOP/PHLPP1 is increased in the hippocampus 24 h after a cardiac arrest mediated global cerebral ischemia in the rat^19^. Similarly, hippocampal SCOP/PHLPP1 levels increased ~2 fold in WTs after a CCI at 7d and 14d post-injury (Figs 2A and 2C), and in the injured cortex **(**Fig. 2B).

### SCOP/PHLPP1 Gene Deletion, MEK activation, and Cognitive Performance after a TBI

**Figure 3:**
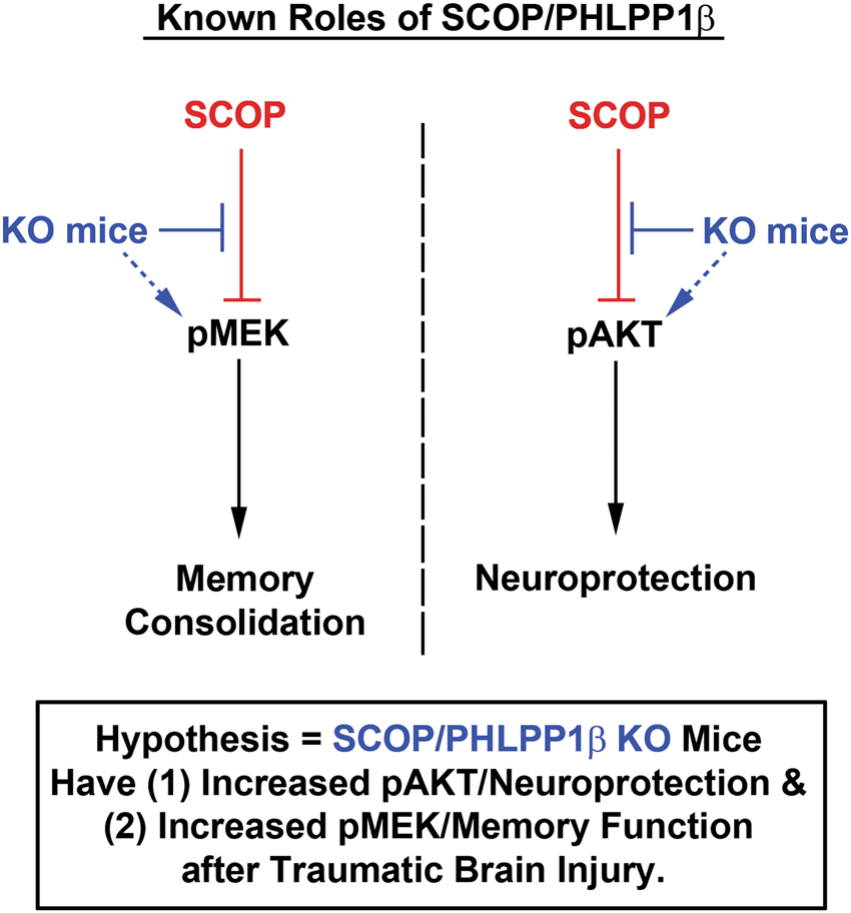
The cell signaling functions of SCOP, and the hypothesized neurological benefits of its therapeutic inhibition in TBI injured KO mice. The diagram illustrates two major cell signaling pathways that are inhibited by SCOP/PHLPP1. MEK activation promotes memory function in the brain. AKT activation promotes cell survival and neuroprotection in the brain. SCOP/PHLPP1 inhibits MEK and AKT, which is predicted to worsen cognitive function and exacerbate neuronal death after TBI. Thus injured KOs are predicted to have better memory performance and histological improvements vs. WT mice.

The primary purpose of this study was to test if inhibiting brain SCOP/PHLPP1 increases tissue survival and improves recovery of memory function after a CCI. Furthermore, we expected increased AKT activation to be associated with improved histology, and increased MEK activation associated with improved memory (Fig. 3). A cell signaling analysis of AKT and MEK was done on tissues isolated at the 7d and 14d post-injury time points because SCOP/PHLPP1 levels were found to be highest in WTs on these days (Fig. 2).

**Figure 4:**
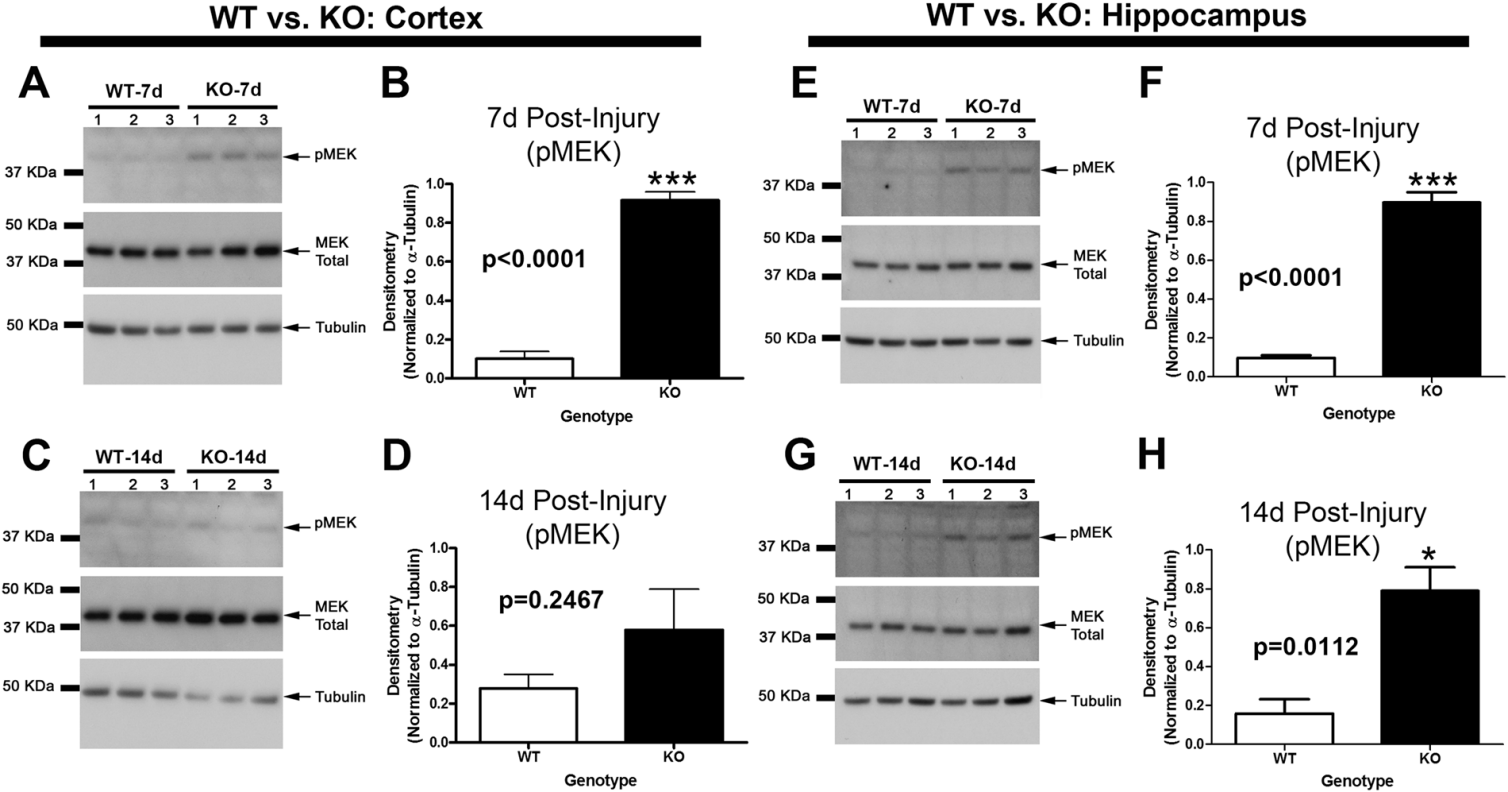
MEK activation is higher in the cortex and hippocampus of KOs. MEK phosphorylation (i.e. activation) in WTs vs. KOs was compared on 7d and 14d post-injury (i.e. key time points at which SCOP/PHLPP1 levels are increased in injured WTs). (**A** and **C**) Western blots show increased cortical pMEK in KOs vs. WTs at 7d but not 14d post-injury. Cropped blots are from the same gel which compared 7d and 14d time points. Each gel probed a different brain region (**B** and **D**) Semi-quantitative densitometry of pMEK levels in the cortex at 7d and 14d post-injury, respectively (n = 3/group). (**E** and **G**) Western blots show increased hippocampal pMEK in KOs vs. WTs at 7d but not 14d post-injury. (**F** and **H**) Semi-quantitative densitometry of pMEK levels in hippocampus at 7d and 14d post-injury, respectively (n = 3/group). The brightness/contrast of MEK blots was optimized in photoshop to enhance clarity; the original unaltered images are available in the Supplementary data. Data were analyzed by unpaired t-test (n = 3/group for all graphs). Data were significant at p < 0.05. Graphs show mean + SEM. White bars indicate WT genotype. Dark bars indicate KO genotype.

In the cortex, MEK phosphorylation (i.e. activation) was significantly increased in KOs vs WTs 7d post-injury (Fig. 4A,B). No differences were observed by 14d post-injury (Fig. 4C,D). In the hippocampus, MEK phosphorylation was significantly increased in KOs vs WTs on 7d post-injury (Fig. 4E,F), and on 14d post-injury (Fig. 4G,H).

**Figure 5:**
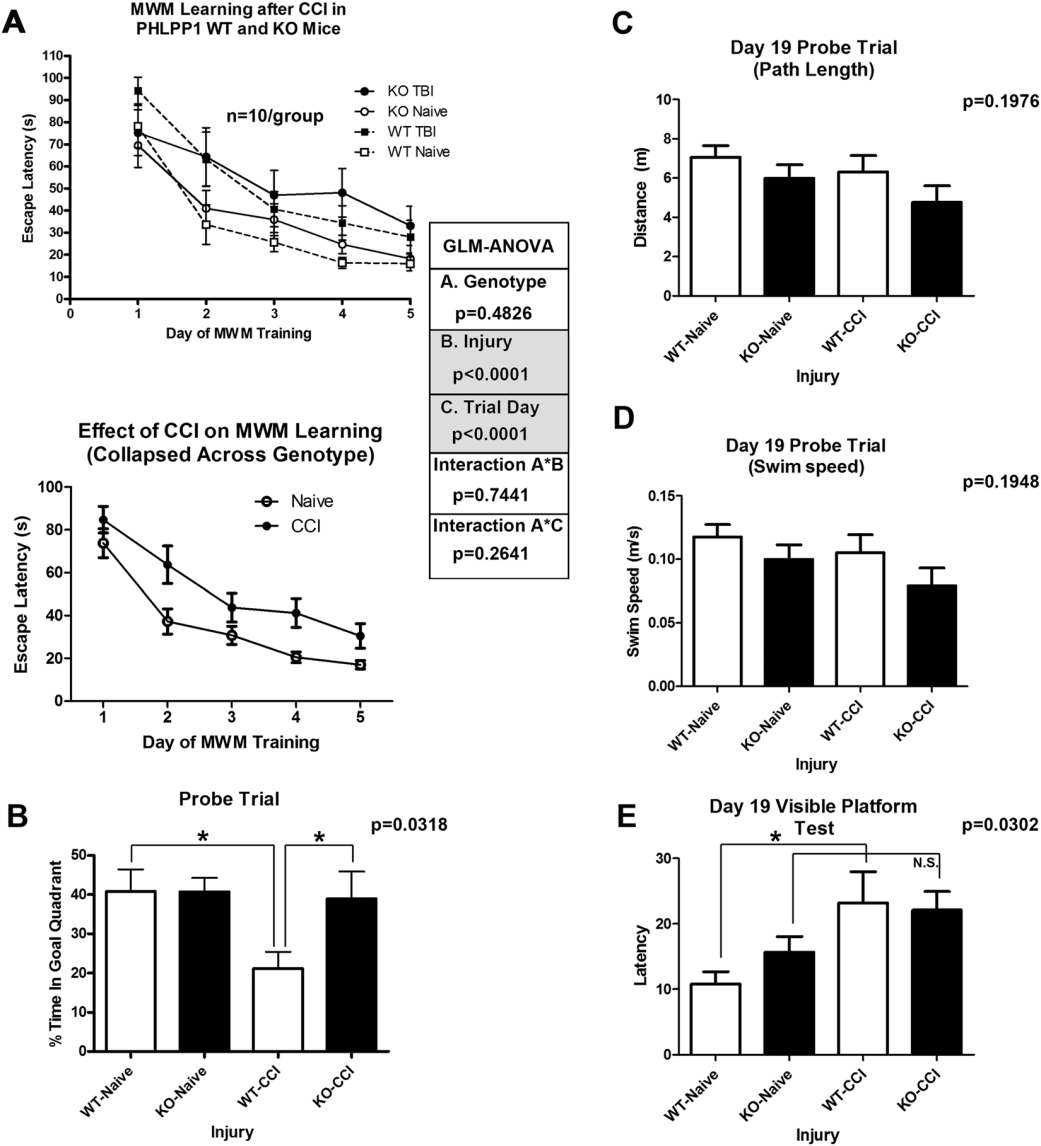
Cognitive Assessment of WT vs. KO mice. Naïve and CCI injured mice were trained on the MWM behavioral task starting 14d post-CCI (n = 10/group). (**A**) Graphs show escape latencies to find the hidden platform across training days (14d–18d); CCI increased escape latencies vs. uninjured naïve (bottom graph collapsed for genotype). (**B**) 19d probe trial test (memory component) of the MWM. Injured KOs spent significantly more time in the goal quadrant vs. injured WTs. There were no significant differences in probe trial (**C**) path length or (**D**) swim speed – indicating normal motor function in all groups. (**E**) Uninjured WTs had significantly better performance on the 19d visible platform test vs. injured WTs. No differences in naïve KOs vs. injured KOs were observed. Learning across days (escape latencies) were analyzed by a three factor GLM ANOVA. Probe trial, path length, swim speed, and visible platform trial, were analyzed by One-Way-ANOVA followed by the Newman-Keuls post hoc test. Data were significant at (*)p < 0.05. Graphs show mean + SEM. White bars indicate WT genotype. Dark bars indicate KO genotype.

We next tested if (1) naïve or (2) CCI-injured KOs vs. WTs had altered learning and memory function. There were no genotype differences in naïve or CCI-injured mice on escape latencies (learning component) across training days (Fig. 5A). Furthermore, probe trial performance (memory component) was equivalent between naïve WTs vs. KOs (Fig. 5B). In contrast, CCI-injured KOs had significantly improved probe trial performance vs. CCI-injured WTs (Fig. 5B). No differences in probe trial path length (Fig. 5C) or swim speed (Fig. 5D) were noted. However, naïve vs. injured WTs had slightly (albeit significantly) better performance on the visible platform control test (Fig. 5E) – possibly due to slightly faster escape latencies in uninjured WTs vs. KOs.

### SCOP/PHLPP1 Gene Deletion, AKT activation, and Tissue Sparing after a TBI

**Figure 6:**
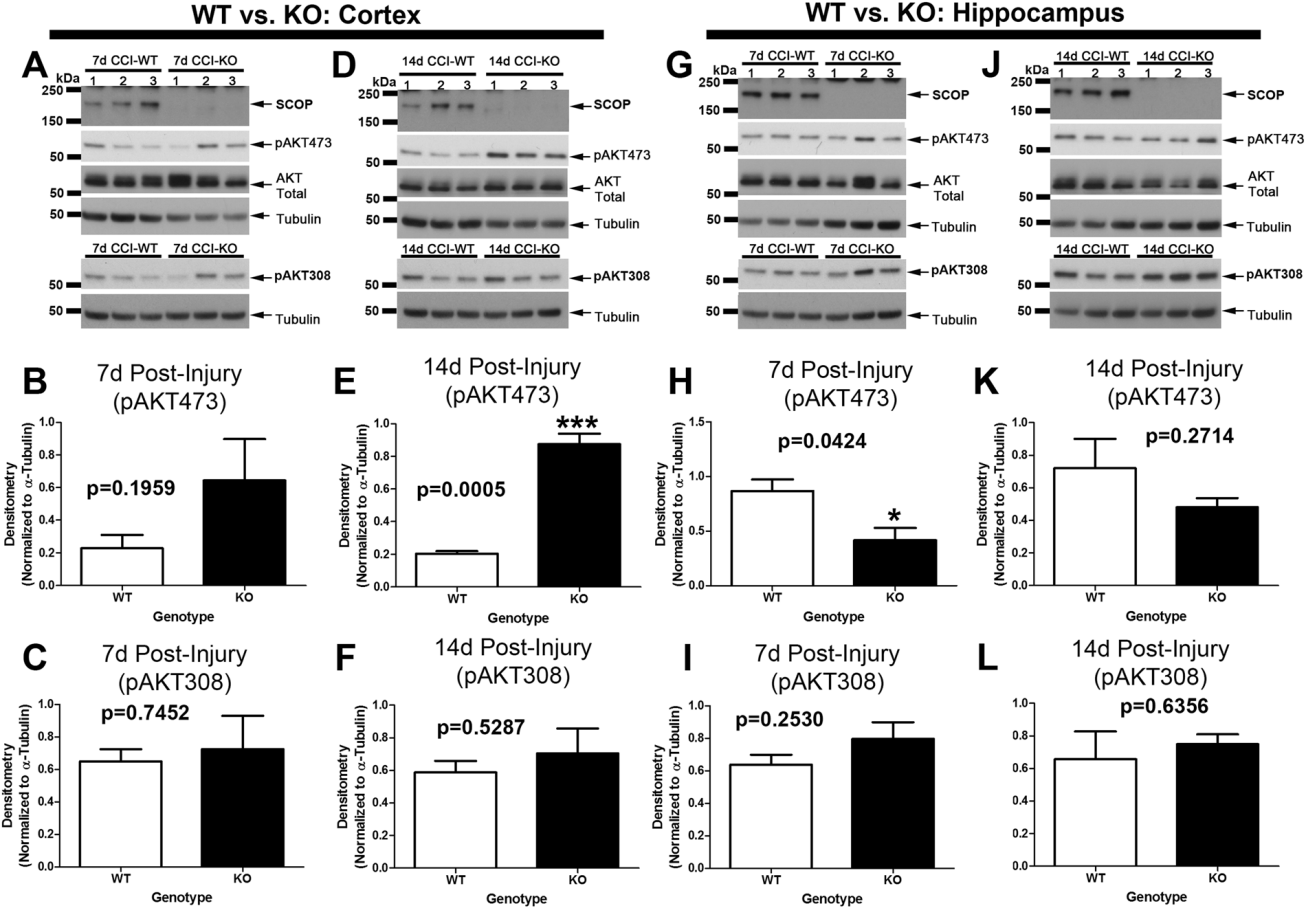
AKT activation is increased in the cortex of KOs 14d post-injury. Phosphorylation levels of AKT activation sites (pAKT473 and pAKT308) were measured in the cortex and hippocampus in WTs vs. KOs at 7d and 14d post-CCI. (**A**–**C**) Cortical pAKT473 and pAKT308 levels were not significantly different by genotype at 7d post-injury. (**D**–**F**) Cortical pAKT473 levels were significantly increased in KOs vs. WTs at 14d post-injury, whereas pAKT308 levels were the same. (**G**–**I**) Hippocampal pAKT473 levels were significantly decreased in KOs vs. WTs at 7d post-injury, whereas pAKT308 levels were the same. (**J–L**) Hippocampal pAKT473 and pAKT308 levels were not significantly different at 14d post-injury. Cropped blots are from multiple gels (see Supplementary data). Tubulin loading controls for pAKT473 vs. pAKT308 densitometry were normalized to their respective gels. Data were analyzed by unpaired t-test (n = 3/group for all graphs). Data were significant at p < 0.05. Graphs show mean + SEM. White bars indicate WT genotype. Dark bars indicate KO genotype.

SCOP/PHLPP1 inhibits pAKT473 phosphorylation (Ser473 is essential for kinase activation)^20^, whereas protein phosphatase 2A (PP2A) inhibits pAKT308 phosphorylation^21^. In the cortex, as expected, pAKT473 levels were increased in KOs vs. WTs 14d post-injury (Fig. 6D,E) but not at 7d post-injury (Fig. 6A,B). Cortical pAKT308 levels were not influenced by genotype (Fig. 6A,C,D and F). In the hippocampus, pAKT473 levels were decreased in KOs vs. WTs 7d post-injury (Fig. 6G,H) but equivalent by 14d post-injury (Fig. 6J,K). Hippocampal pAKT308 levels were not influenced by genotype (Fig. 6G,I,J and L).

**Figure 7:**
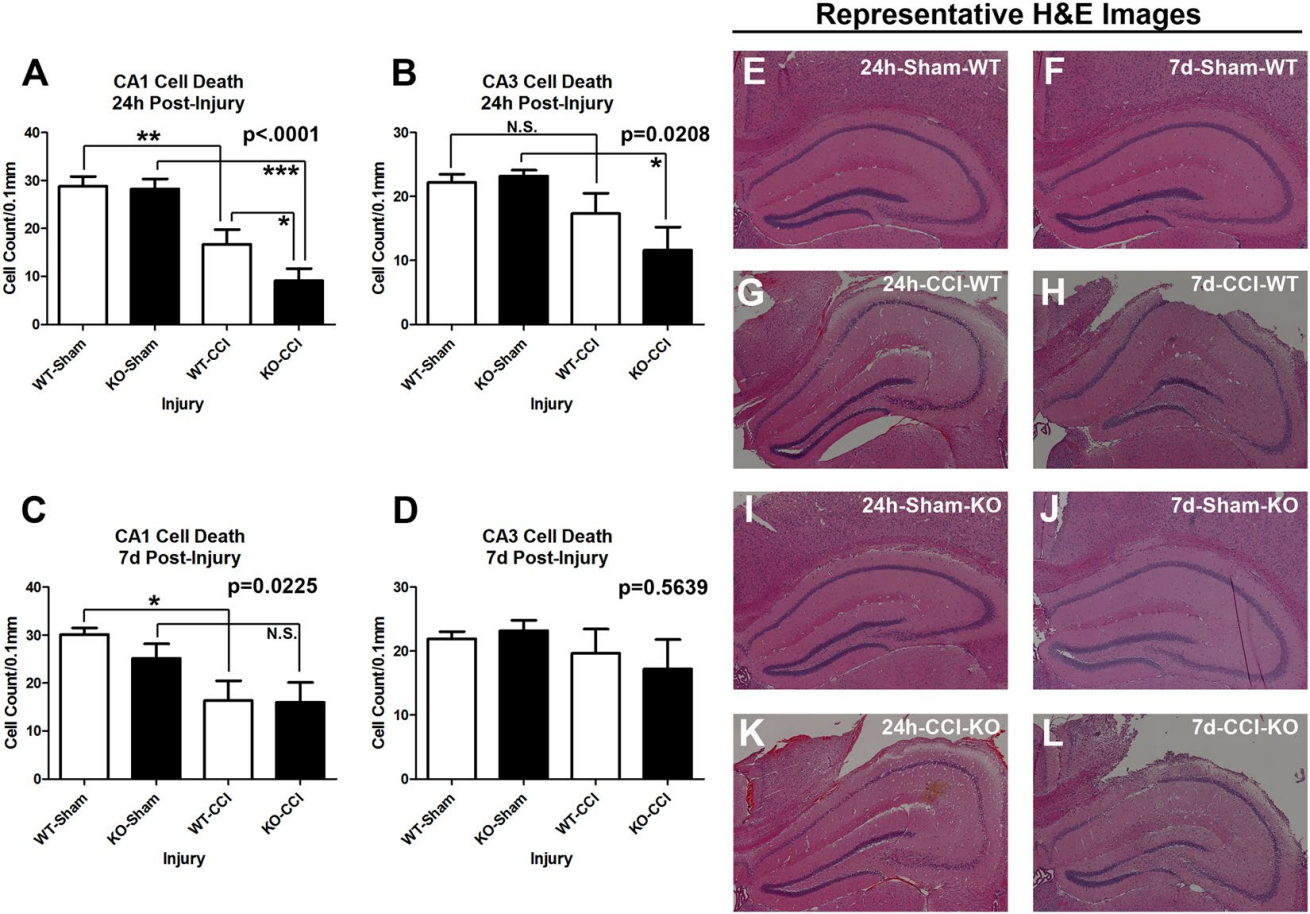
Acute hippocampal survival in CA1/CA3 is worse in KOs vs. WTs after a TBI. Brains were collected 24 h and 7d after CCI injury for histological analysis (n = 5/group). (**A**) Hippocampal CA1 cell counts 24 h after a CCI. (**B**) Hippocampal CA3 cell counts 24 h after a CCI. (**C**) Hippocampal CA1 cell counts 7dh after a CCI. (**D**) Hippocampal CA3 cell counts 7d after a CCI. (**E**–**L**) Representative images of H&E stained brain sections. Data were analyzed by One-Way-ANOVA followed by the Newman-Keuls post hoc test. Data were significant at p < 0.05. Graphs show mean + SEM. White bars indicate WT genotype. Dark bars indicate KO genotype.

**Figure 8:**
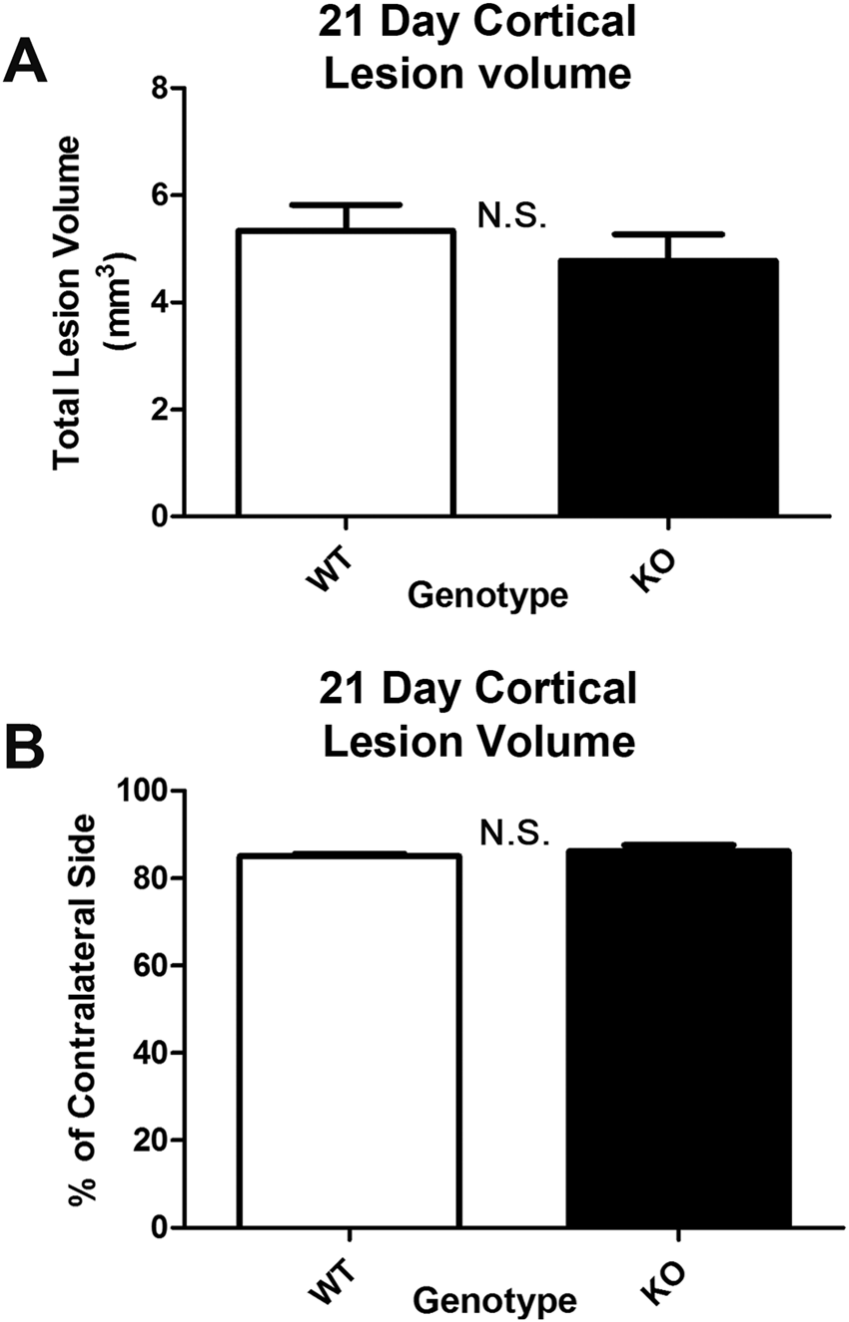
21d lesion volume after a CCI in WT vs. KOs. MWM trained mice were sacrificed on 21d post-CCI for volumetric analysis of cortical tissue. (**A**) Total lesion volume in the ipsilateral cortex. (**B**) Lesion volume normalized as a percent of the contralateral side. Genotype had no effect on cortical lesion volume. Data were analyzed by unpaired t-test. Data were significant at p < 0.05. Graphs show mean + SEM. White bars indicate WT genotype. Dark bars indicate KO genotype. N.S. indicates “not significant”.

Next we measured hippocampal survival by our standard protocol in CCI^12^. As expected, a CCI injury significantly decreased the number of live cells in the CA1 stratum pyramidale vs. uninjured shams across genotypes (Fig. 7A). CA1 cell death was exacerbated in SCOP/PHLPP1 KOs 24 h post-injury vs. WTs (Fig. 7A). Furthermore, SCOP/PHLPP1 deletion appeared to exacerbate 24 h post-injury in the CA3 (Fig. 7B). In contrast, by 7d post-injury, WTs appeared to have worse CA1 survival (Fig. 7C); however, that result may be influenced by the observation that KO shams had slightly worse damage vs. WT shams (i.e. the craniotomy alone induces a mild injury). Increased damage in KO shams would decrease the effect size to observe a difference vs. CCI-injured KOs (Fig. 7C). No genotype differences in 7d CA3 cell counts were observed (Fig. 7D). Representative images of H&E stained sections are illustrated (Fig. 7E–L). Cortical lesion volume 21d post-injury did not differ between genotypes (Fig. 8A). Also, there were no differences after normalizing the ipsilateral lesion volume to the contralateral side (Fig. 8B).

**Figure 9:**
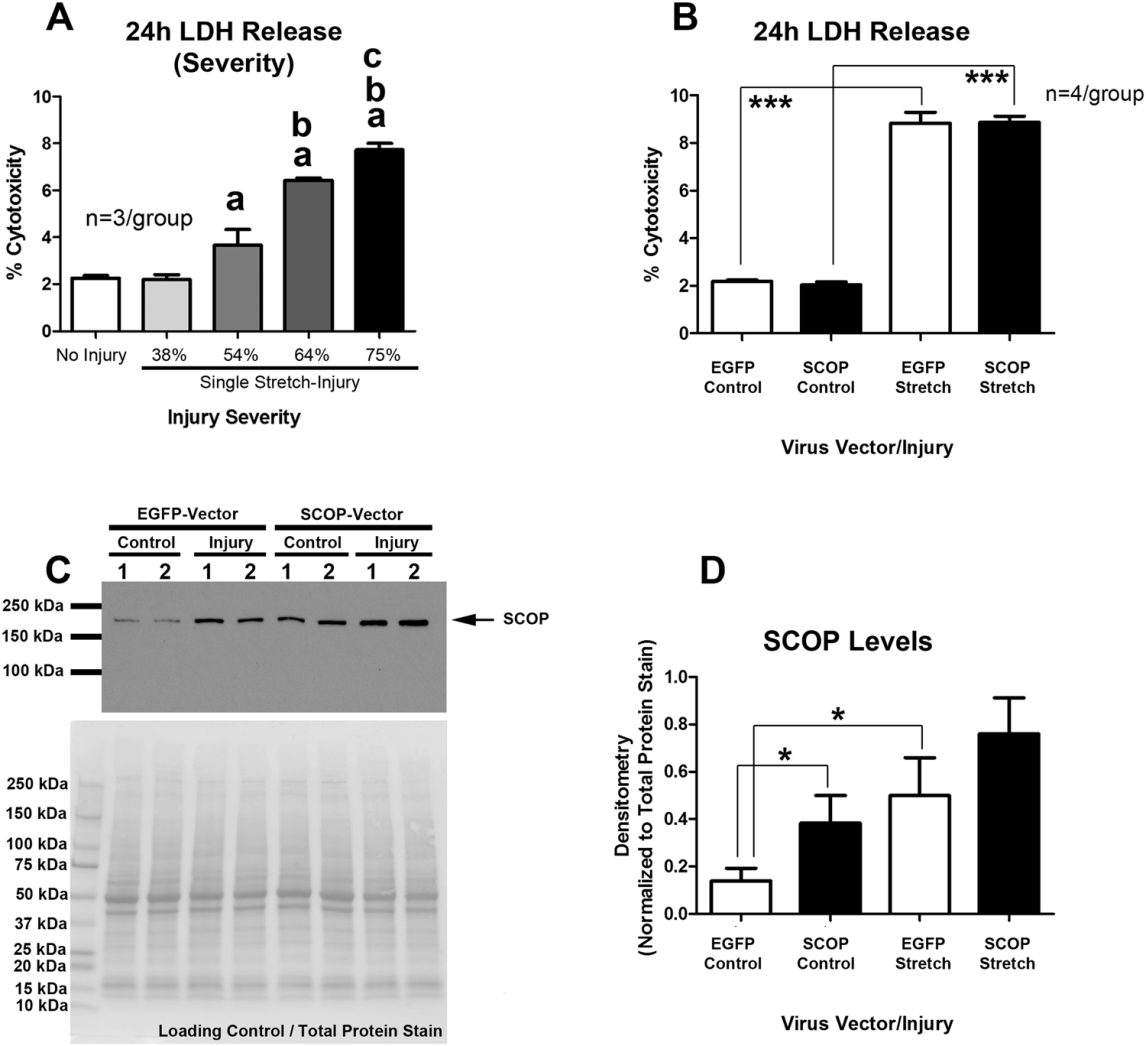
Effect of SCOP/PHLPP1 overexpression on cell survival in rat cortical neurons subjected to a mechanical stretch-injury. Rat primary cortical neurons were grown on deformable Silastic membranes and injured on DIV9 by a 50 ms stretch-injury. (**A**) Assessment of 24 h LDH release (i.e. cell death) with increasing injury severity in non-transduced neurons (i.e. increased magnitudes of % membrane deformation) (n = 3/group). (**B**) The effect SCOP/PHLPP1 overexpression on 24 h LDH levels in neurons subjected to a severe stretch-injury (~75% membrane deformation). (**C**) A representative Western blot showing SCOP/PHLPP1 levels in uninjured vs. stretch-injured neurons transduced with either a control vector (EGFP) or experimental vector (SCOP/PHLPP1). Total protein stain of PVDF membrane shows equal protein loading across groups. (**D**) Semi-quantitative densitometry of SCOP/PHLPP1 levels (n = 4/group). Data were analyzed by One-Way-ANOVA followed by Newman-Keuls post hoc test. The log transformation of raw LDH values in Panel D was used for statistical analysis. Cropped Western blot is from a single gel. Data were significant (*) at p < 0.05. (a) = significantly different vs. No Injury, (b) = significantly different vs. 54% stretch, (c) = significantly different vs. 64% stretch. Graphs show mean + SEM. White bars in panels B/D indicate EGFP genotype. Dark bars in panels B/D indicate SCOP/PHLPP1 overexpression.

The evolution of secondary injury (e.g. altered cerebral blood flow and brain edema) after a TBI is influenced by extra-cerebral factors such as MAP, which was increased in KOs vs. WTs after a CCI. To investigate SCOP/PHLPP1 mediated neuronal cell death in a pure system we used our *in vitro* model of mechanical-stretch injury. First we identified the level of injury (single-stretch) which yielded robust neuronal death 24 h later (Fig. 9A). A 75% biaxial strain induced pronounced 24 h LDH release vs. controls (Fig. 9A). Neurons were then transduced with lentivirus to deliver either a control vector (EGFP) or a vector which overexpresses recombinant mouse SCOP/PHLPP1 (Fig. 9C). SCOP overexpression levels were ~2 fold higher in uninjured DIV10 neurons transduced with a SCOP overexpressing vector vs. uninjured neurons transduced with a control vector. Overexpression of SCOP/PHLPP1 had no effect on 24 h cell death (i.e. LDH release) after a stretch-injury (Fig. 9B). However, mechanical stretch-injury led to an increase in endogenous SCOP/PHLPP1 levels 24 h later (Fig. 9C,D).

## Discussion

### Altered Physiology in SCOP/PHLPP1 KO Mice

Whole-body SCOP/PHLPP1 KO mice have been studied in numerous other diseases including in stroke^7,10,15–18^. A key unanticipated finding in this study is that SCOP/PHLPP1 KOs exhibit alterations in baseline and post-injury physiology which may be important. This includes alterations in MAP, arterial blood gases, pH, bicarbonate, and osmolarity (Fig. 1D).

Interestingly, KOs resisted the acute reduction in MAP that is seen after a CCI^13^, and which can also occur after a TBI in humans^22^. Genotype-dependent differences in MAP emerged ~10–15 min post-injury, and persisted across the full monitoring period 60 min after a CCI. The consequence of an elevated post-injury MAP in KOs is unknown but high or low MAP thresholds in the comprised brain after a TBI influence lesion volume, hippocampal neuronal death, tissue oxygenation, and edema^23–26^ Kroppenstedt and colleagues reported that, 4 h after a CCI in rats, a brief 30 min 20 mmHG reduction in MAP significantly increased lesion volume 28 h later^25^. Thus, based on the magnitude (~17 mmHG) and duration (~45–50 min) of the difference in MAP seen in our study, we expected WTs to have worse histological outcomes. Contrary to that prediction, 24 h CA1/CA3 cell death was less severe in WTs vs. KOs. Also, there was no genotype difference in 21d lesion volume. Therefore, our histological assessment does not suggest that WTs had greater secondary injury vs. KOs. Moreover, it is unclear why 24 h hippocampal injury is slightly worse in KOs given that they had a normal MAP post-injury. One possible explanation is that inhibition of the SCOP–regulated mechanism(s) that in turn mediate a stable blood pressure (refractory to TBI), may also be associated with a broader (unidentified) pathology which negates the potential benefits of raised MAP to reduce injury.

More experiments outside the scope of this study are needed to investigate the nature of MAP changes in KOs, and to address if/how they affect neurological outcomes after a CCI. The difference in MAP in KOs vs. WTs would be expected to yield clinically meaningful changes in cerebral perfusion pressure (CPP)^25,26^. Thus, additional physiological parameters that need to be assessed in WTs vs. KOs after a CCI include cerebral blood flow (e.g. via arterial spin labeling MRI)^13^, intracranial pressure (via ICP monitoring)^27^, and analysis of pressure autoregulation^28^. Furthermore, a comparison of total body vs. conditional (brain specific) SCOP/PHLPP1 KOs would help to address if cognitive benefits seen here were due to central and/or peripheral mechanisms. Using an *in vitro* neuronal stretch-injury model we found that SCOP/PHLPP1 overexpression had no effect on 24 h cell death, which supports the idea that altered physiology in KOs may partly mediate cognitive benefits. Finally, studies using remote telemetry may unmask small differences in MAP at various points during the circadian cycle of KOs, and also to track the time at which injury induced changes in MAP returns to baseline in WTs.

In addition to MAP, differences in PaCO_2_, PaO_2_, pH, bicarbonate, base excess, and osmolality were also notable, and the effect of each on outcomes post-injury remain undefined. Genotype differences in PaCO_2_ and PaO_2_ would represent clinically meaningful differences, while the difference in osmolality was quite small. Elevated PaCO_2_ causes vasodilation of the cerebral vasculature, which increases cerebral blood flow/CBF^29^. Normal baseline PaCO_2_ levels in isoflurane anesthetized mice range from ~25–28 mmHG^12,30^. High PaCO_2_ levels in KOs were below the threshold of hypercapnia (i.e. <45 mmHG)^31^. However, in the compromised brain, even borderline hypercapnia may be detrimental. The main concern, particularly in the setting of severe TBI, is that hypercapnia may contribute to increased cerebral blood volume, hyperemia, and promote intracranial hypertension^32,33^. Thus, it is possible that secondary injury mechanisms related to high PaCO_2_ exacerbated hippocampal damage in KOs. However, additional studies are needed to critically evaluate these assumptions, and also to consider the impact of high PaCO_2_ in concert with other overlapping physiological changes such as MAP. We also recognize that the specific mechanism(s) underlying the observed physiological differences remain to be identified.

A few limitations must be addressed. (1) Physiology was measured under isoflurane/nitrous oxide anesthesia after preparatory surgery. Thus, physiological differences in KOs may not manifest, or be as severe, under normal awake conditions (which was not investigated here). It was interesting that the KO phenotype appeared to blunt the reduction in PaCO_2_ and increase in pH observed in the WT after preparatory surgery (vascular catheter placement and craniotomy) and prior to TBI. This may have resulted from either a blunted stress response, and/or an alteration in anesthetic MAC in the KO vs. WT. The other blood gas findings (decreased PaO_2_, reduction in bicarbonate, and BE, in the KO vs. WT) could logically represent secondary and compensatory effects of the respiratory alkalosis seen in the WT vs. KO. Curiously, these findings were evident at baseline prior to TBI despite no difference in MAP and no difference in respiratory rate between genotypes. However, even if differences in the response to anesthesia or surgical stress precipitated these effects, the differential response is an important finding that merits consideration. It strongly indicates that physiological parameters need to be carefully assessed in studies investigating either SCOP/PHLPP1 mutant strains or drugs targeting this pathway, which would act systemically much like whole body KO here and potentially under conditions of anesthesia in brain injured patients. (2) The genetic background of our mice (B6129SF1/J) is slightly different vs. past studies that used similar whole body KOs on a B6X129 background^15^. Thus, we cannot rule out the possibility that some of the new findings reported here are related to the interaction between genotype and genetic background of our mice. However, SCOP/PHLPP1 inhibitors, if developed for human clinical trials, would ultimately face much greater biological diversity in patient backgrounds.

### MEK Activation and Memory Function after a TBI in KOs vs. WTs

MEK activation promotes memory function, and SCOP/PHLPP1 inhibits MEK^3^. Also, MEK inhibitors applied to the brain specifically impair memory function but do not impede learning^4,34,35^. Here we found that SCOP/PHLPP1 levels increased in the hippocampus and cortex after a CCI in WTs at 7d and 14d post-injury. Therefore, we anticipated that (a) KOs would have higher MEK activation in the injured brain at these time points, and (b) have improved memory performance after a CCI but no improvements in learning. We confirmed these predictions but more studies are needed to verify that increased MEK was responsible for cognitive benefits. Specifically, future studies involving injection of MEK inhibitors directly into the KO brain (regionally) are needed to clarify if improved memory performance after a CCI in KOs was due to increased MEK activation in the cortex, the hippocampus, or possibly due to upregulation in both regions. Alternatively, other signaling pathways regulated by SCOP may play a key role in mediating cognitive improvements.

Prior work suggests that SCOP/PHLPP1 disturbances in either direction (overexpression or knockdown) in the hippocampus can impair memory function^6^. Specifically, (1) overexpression of SCOP/PHLPP1 in the mouse forebrain impaired memory on a novel object recognition task^5^. Alternately, (2) conditional KO of SCOP/PHLPP1 in the mouse neocortex/hippocampus also impaired memory on a novel object recognition task^6^. (3) In whole body SCOP/PHLPP1 KOs, baseline memory performance is impaired on an object location memory (OLM) and recognition memory (ORM) task^7^, but (4) is unimpaired on a fear-conditioning based memory task^7^. Thus, the type of memory task used to assess cognition in SCOP/PHLPP1 KOs is an important factor. Here we report the first analysis of SCOP/PHLPP1 KOs on the MWM. Baseline learning and memory performance did not differ in naïve WTs vs. KOs using our standard protocol^36^. After a CCI, memory function was improved in KOs. We chose the MWM because it is the gold standard in pre-clinical TBI research - due to high correlation between functional deficits detected by this paradigm and histological lesion volume^37^. Furthermore, MEK showed the largest fold change in KOs compared to other signaling targets examined, and the MWM can detect MEK disturbances with high sensitivity (even on a 1d training protocol)^4^. In future studies, a battery of behavioral tests (e.g. open field maze/anxiety^37^, reversal MWM/memory extinction^38^, and attentional set-shifting/frontal lobe function)^39^ may uncover additional benefits or disturbances induced by SCOP/PHLPP1 KO after a CCI.

### AKT Activation and Histology after a TBI in KOs vs. WTs

We expected to observe widespread brain tissue sparing effects in KOs after a TBI. In the cortex, lesion volume was equivalent in injured KOs vs. WTs (despite increased pAKT473 levels in KOs). Also, rat cortical neurons overexpressing recombinant SCOP had similar survival outcomes after a stretch-injury *in vitro*. Together these findings suggest that increased SCOP/PHLPP1 levels observed in the cortex after a TBI may play a minor role in neuronal survival. Furthermore, deletion of SCOP/PHLPP1 in the hippocampus worsened outcome. KOs had decreased CA1 cell survival 24 h post-injury. Levels of pAKT473 were also slightly decreased in the hippocampus of injured KOs. These findings suggest that, contrary to our hypothesis, SCOP/PHLPP1 may promote hippocampal survival in the acute post-injury period. Our prior reports offer some insight as to a possible mechanism; selectively inhibiting SCOP by lentivirus mediated RNAi in primary hippocampal neurons decreased pAKT473/pAKT308 levels and increased pro-apoptotic FOXO3a levels^40^. However, more studies are needed to elucidate potential signaling mechanisms involved in SCOP mediated neuroprotection – which seems to occur selectively in the hippocampus. Several limitations of AKT studies in this report must be acknowledged. (1) AKT activation increases necroptosis mediated cell death after a TBI^41–43^. Thus, future studies are needed to test if RIPK pathways are increased in KOs after a TBI, and if it contributed to the lack of neuroprotection seen here. (2) AKT activation is also regulated by pAKTSer477/pAKTThr479 phosphorylation, and by binding to carboxyl-terminal modulator protein (CTMP), which were not studied here but could also play an important role in KOs^44,45^. Notably, CTMP which blocks pAKT473/pAKT308 phosphorylation (i.e. the sites investigated here) is increased in the brain after CCI-TBI in mice^46^.

The effect of genotype on pAKT473 was region and time -dependent. Hippocampal pAKT473 levels were significantly decreased 7d post-injury, whereas cortical pAKT473 levels were significantly increased 14d post-injury. Additional studies are needed to elucidate the molecular underpinnings to explain this discrepancy. We speculate that regional differences in the distribution of AKT isoforms may play a role. It was recently demonstrated that protein levels of AKT1 and AKT3 isoforms are abundant across hippocampal sub-regions^47^. In contrast, AKT2 protein expression is low in the CA1/CA3^47^. Importantly, AKT2 is the major isoform dephosphorylated by SCOP/PHLPP1^48^. Our anti-pAKT473 antibody does not distinguish AKT1 vs. AKT2 vs. AKT3 (i.e. due to shared sequence similarity in all isoforms in the region surrounding the serine 473 site). Thus, hippocampal vs. cortical AKT2 levels may differ before and after CCI injury, and have contributed to the unique regional and temporal pAKT473 expression patterns which we observed in SCOP/PHLPP1 KOs.

## Conclusion

In summary, here we tested if SCOP/PHLPP1 gene deletion improves behavioral outcomes in mice given a TBI. Specifically, we observed no evidence on tissue sparing effects in KOs vs. WTs. However, we did observe marked benefits on the memory component of the MWM behavioral test. The latter finding is in agreement with observations of increased MEK phosphorylation. More studies are warranted to investigate if SCOP/PHLPP1 inhibition is a robust and/or translatable strategy to enhance cognitive recovery in the setting of acute brain injury. Future studies are also warranted to expand upon our initial analysis of acute physiology in KOs, and to determine if any of the observed differences also occur chronically in awake mice. The underpinnings of physiological changes in KOs remains unknown but our results on MAP and blood chemistry raise concerns germane to the pleotropic roles of SCOP/PHLPP1 and its regulation of biological processes in other organs that have yet to be discovered.

## Methods

### Antibodies

Anti-SCOP/PHLPP1 (COSMO Bio; Carlsbad, CA, USA), anti-pAKT473, anti-pAKT308, anti-AKT Total, anti-pMEK, anti-MEK Total, anti-α-Tubulin (Cell Signaling Technology; Danvers, MA, USA).

### Animals

Animal work was approved by the Institutional Animal Care and Use Committee (IACUC) of the University of Pittsburgh and by the Animal Care and Use Review Office (ACURO) of the US Army/department of defense. All experiments were performed in accordance with relevant guidelines and regulations. Euthanasia protocols follow guidelines established by the America Medical Veterinary Association Guideline for Euthanasia. Heterozygous (+/−) SCOP/PHLPP1 wt and KO mice were provided as a generous gift from Dr. Tianyan Gao at University of Kentucky. The SCOP/PHLPP1 targeting strategy and PCR genotyping was described previously^15^. Mice were re-derived by The Jackson Laboratories (Bar Harbor, ME, USA) as we reported^49^. Breeding/genotyping was performed at Safar Center for Resuscitation Research. Male WT/KO mice were injured at ~12 wk of age, and littermates were used in all experiments. Timed pregnant (E16–17) Sprague Dawley rats (Charles River) were used for *in vitro* primary cortical neuron cultures studies.

### TBI Model

Briefly, all CCI-injured or sham mice were anesthetized with 4% isoflurane (induction) and 1–2% (maintenance) in 70% Nitrous Oxide (N_2_O)/30%O_2_. Mice were given moderate CCI using our standard protocol^50^. Temperature was maintained at 37 °C. The head was shaved and prepped with betadine. Skin above the target injury site was opened and a small 5 mm craniotomy introduced for access to the left parietal cortex. Mice were given CCI using a pneumatic impactor device, at an injury level previously demonstrated by our group to induce hippocampal damage (velocity 6 m/s; depth 1.2 mm)^50^. The incision site was sutured, and mice were immediately weaned from anesthesia then allowed to recover on supplemental oxygen for 30 min to mimic clinical care before returning to the housing facility. Animals were randomized by the age at which they became available to enter the study.

### Physiology

A separate cohort of mice (n = 5/genotype) were used to assess genotype differences in mean arterial blood pressure (MAP; Fig. 1A,B), arterial blood gases, and blood chemistries (Fig. 1D). Mice were anesthetized with 4% isoflurane (induction) and 1–2% (maintenance) in 70% Nitrous Oxide (N_2_O)/30%O_2_, and switched to room air prior to a CCI. Mice were maintained on 1–2% anesthesia throughout the 1 h post-injury observation period. Arterial blood was collected at baseline and after CCI via femoral arterial catheter (70 μL aliquots). Baseline samples were collected 15 min before CCI (under 1–2% isoflurane), 5 min pre-CCI, 10 min post-CCI, and 60 min post-CCI. Whole blood was immediately analyzed on an ABL90 Radiometer Blood Gas Machine (Radiometer Medical ApS; Copenhagen, Denmark). *Respiratory Rate*: A separate cohort of mice (n = 5/genotype) were used to measure respiratory rate. Naive isoflurane anesthetized mice were placed in a MouseOx apparatus (Starr Life Sciences Corp; Oakmont, PA, USA) and respiratory rate recorded for 20 min under FiO2 of 33% 66%N_2_O)/O_2_ balance) immediately followed by 65 min in room air. Mice were returned to standard housing upon conclusion of these measurements.

### Assessment of Learning and Memory

MWM testing was performed as previously described by our group^51^. A total of n = 41 mice were used for behavioral analysis (i.e. n = 10/group; 1 animal died). The same mice were also used for 21d contusion volume analysis as described below. Briefly, training was done 14–18d post-TBI in a 100 cm diameter pool (60 cm high) and water temperature set to ~24 °C. Stationary extra-maze cues are visible around the 2.5 × 2.5 diameter room. Swim path was recorded via a video tracking system (AnyMAZE, Stoelting, Inc.; Wood Dale, IL, USA). Each training trial to find the hidden platform (10 cm diameter/clear plastic) consisted of four randomized placements in the pool. Mice were allowed a maximum of 120 sec to find the submerged platform and given a 4 min rest in a 37 °C incubator between trials. MWM performance was quantified as latency to find the hidden platform. On post-injury day 19 a single 60 s visible platform test was done (latency to find the platform) and 60 s probe trial (platform removed). Probe trial is calculated as percent time spent in target quadrant. Investigators were blinded to genotype/injury groups for behavioral assessment.

### Histology and Contusion Volume

Histology and contusion volume were assessed as previously described^12^. A total of 40 mice were used for H&E CA1/CA3 cell counts (i.e. n = 5/group). Briefly, mice were perfused with heparinized cold saline followed by 10% formalin fix under anesthesia. Brains were post-fixed for an additional 72 h in 10% formalin. 3 mm coronal brain sections were made and embedded in paraffin wax. Embedded brains were cut using a microtome to make 5 µM sections. Sections were mounted on glass slides and stained with Hematoxylin and Eosin (H&E; Thermo Scientific – Shandon; Pittsburgh, PA, USA) to quantify CA1/CA3 cell death in hippocampus. Images were captured on a Nikon Eclipse 90i microscope (Nikon; Melville, NY, USA). For volumetric analysis of contusion size, mice were perfusion fixed as described above and entire brains cryosectioned; 10 µm sections were made every 0.5 mm. Brain area and lesion volume were analyzed using the program morphometric image analysis (MCID; Ontario, Canada). The technician quantifying CA1/CA3 cell counts and contusion volume was blind to genotype/injury groups.

### Primary Neuron Culture

Neurons were cultured using our standard protocol as previously described^49^. Briefly, brains were collected from E16–17 rat embryos and dissected in ice-cold hanks balanced salt solution (HBSS). Cortices were trypsinized, triturated, and cell pellet resuspended in Neurocult®/SM1 supplement (containing 25 µM glutamic acid and 0.5 mM L glutamine). Neurons were counted on a Cellometer and seeded onto Bioflex plates (Flexcell International Corp.; Burlington, NC, USA), coated with 0.5 mg/mL Matrigel. Cells were plated at a density of ~1.5 × 10^6^ per well. Cultures were maintained by ½ media exchange with BrainPhys®/SM1 media on DIV 5 and 8.

### High-Titer Lentivirus Production

Lentivirus production was done as described by our group^52^. BSL-2 work was approved by the University of Pittsburgh Institutional Biosafety Committee. Expression plasmids were purchased from GeneCopoeia (Rockville, MD, USA). The control vector is an EGFP expression transcript in the pReceiver-Lv156 lentiviral plasmid system (Cat# Ex-EGFP-Lv156). The experimental vector is the mouse-specific SCOP/PHLPP1 open reading frame sequence in the Lv156 lentiviral plasmid system (Cat# Mm25576, Accession #NM_133831.3). In brief, purchased expression vectors were maxi-prepped, and concentrations of high-purity plasmids measured on a UV spectrophotometer. Expression plasmids and packaging plasmids (purchased from GeneCopoeia) were mixed in basal Opti-MEM culture media, combined with EndoFectin, diluted into Opti-MEM/10%FBS/Pen-Strep, and transduced on HEK293ta cells in T225 culture flasks. 12 h after transduction, the medium was removed and replaced with ~18 mL fresh maintenance media per flask. Virus containing supernatant was collected at ~36 h and ~48 h post-transduction, pooled, passed through a 0.45 µM filter top system, concentrated in Centricon® Plus-70 Centrifugal Filter Units, further concentrated by ultra-centrifugation (1.5 h/24,000 rpm/4 °C), the pellets resuspended in 200 µL basal Opti-MEM, and left overnight in a 4 °C refrigerator. The following day concentrated virus was aliquoted into sterile screw-cap tubes and stored at −80 °C. Viral titer was measured using the Quick Titer™ Lentivirus Titer Kit (Cell Biolabs Inc; San Diego, CA, USA). Neurons were transduced on the day of plating (DIV0) with a multiplicity of infection of 30 (MOI 30).

### *In Vitro* Mechanical Stretch Injury

Mechanical stretch-injury was done as described previously^53^. In brief, on DIV9, ~750 µL of conditioned media (C.M.) was collected from each well, pooled but separated by viral manipulation, and diluted with an equal volume of fresh BrainPhys®/SM1 to generate treatment media (i.e. 50% C.M.). The remaining media was aspirated off wells, replaced with 1 mL 50% C.M., subjected to a stretch-injury using the CIC-II device (Custom Design & Fabrication Inc.; Sandston, VA, USA), and culture plates returned to a 37 °C 5% CO_2_ incubator for 24 h. After collection of medium for LDH analysis, cells were washed 1X with PBS, and left on ice for ~1 h in Cell Recovery Solution® to dissolve Matrigel. Neurons were scraped off the Silastic membranes, transferred into a 1.5 mL tube, centrifuged for 5 min/4 °C/200 g, resuspended 1X in PBS, centrifuged again for 5 min/4 °C/200 g, and proteins extracts made by re-suspending cell pellet in ~65 µL RIPA buffer (with EDTA/Protease/Phosphatase Inhibitors). The % biaxial stretch of the Silastic membrane is indirectly measured by assessing the peak pressure achieved inside the well during the 50 ms nitrogen burst (measured by a pressure transducer connected to the CIC-II device). The average peak pressures (n = 3/group) in Fig. 9A were 2.73 ± 0.058 (~38% stretch), 4.10 ± 0.173 (~54% stretch), 5.07 ± 0.115 (~64% stretch), and 5.57 ± 0.058 (~75% stretch). The average peak pressures (n = 4/group) in Fig. 9B for EGFP controls were 5.58 ± 0.05 (~75% stretch) and for SCOP/PHLPP1 overexpression were 5.58 ± 0.05 (~75% stretch).

### Lactate Dehydrogenase (LDH) Cell Death Assay

LDH analysis was performed as described by our group^54^. Treatment media (50% C.M.) was saved as the negative control. 400 µL of media was collected from neurons 24 h after mechanical-stretch injury, from uninjured controls, and from uninjured controls lysed 24 later to obtain maximum LDH values. Media was centrifuged ~10,000 g/5 mins/4 °C to remove debris. Samples were run in triplicate. LDH levels were analyzed using the LDH Cytotoxicity Assay Kit II (Abcam; Cambridge, MA, USA). The percent cytotoxicity was calculated by the equation:

$$\begin{array}{rcl} \% \,{\rm{cytotoxicity}} & = & ({\rm{sample}}\,{\rm{value}}-{\rm{negative}}\,{\rm{control}})/({\rm{maximum}}\,{\rm{LDH}}\,{\rm{control}}\\  &  & -\,{\rm{negative}}\,{\rm{control}})\times 100.\end{array}$$

### Western Blot Analysis

Proteins were analyzed as previously described by our group^19^. 24 mice were used for *ex vivo* brain tissue Western blot studies (n = 3/group). Briefly, cells/tissues were pulse-sonicated 20–30 s on ice in RIPA buffer plus inhibitors. Homogenates were spun in an ultracentrifuge at 4 °C/10 min/16,000 g. Total protein extracts (supernatants) were analyzed by BCA (PIERCE; Rockford, IL, USA) to estimate protein concentrations. Samples (10–20 µg/well) were loaded onto pre-cast 4–15% gradient SDS-PAGE gels (BioRad; Hercules, CA, USA). Protein was transferred to PVDF membrane (GE Healthcare; *Hybond-P*; Pittsburgh, PA, USA). Primary antibodies were diluted in tris-buffered saline plus tween-20 (TBS-T) with 7.5% milk, and incubated on membranes overnight. Membranes were washed with TBS, incubated 2 h with secondary antibodies, washed with TBS, treated with ECL-II Western blot detection reagent (Fisher Scientific; Pittsburgh, PA, USA), exposed to films in a dark room, developed, and images collected via a scanner. Blots were stripped and re-probed with subsequent antibodies using methods described above. The order in which targets were detected is indicated in the Supplementary data. Images for figures were compiled in Photoshop (Adobe; San Jose, CA, USA).

### Statistics

Physiology data were analyzed by Two-Way Repeated Measures ANOVA and MWM latency by three factors GLM ANOVA in NSCC Statistical Software. Densitometic analysis of SCOP/PHLPP1 western blot data, CA1/CA3 Cell death, Day 19 MWM probe trial, path length, swim speed, visible platform, and *in vitro* LDH release (transformed values) were analyzed by One-Way ANOVA. Densitometic analysis of AKT/MEK, analysis of ipsilateral total lesion volume, and % contralateral side were analyzed by unpaired T-test in GraphPad Prism Software (GraphPad Prism; La Jolla, CA, USA). Post-hoc differences for Two-way Repeat Measures ANOVA were analyzed by Bonferroni. Post-hoc differences for One-Way ANOVA were analyzed by Newman-Keuls. Data were significant at p < 0.05. Graphs show mean + SEM.

### Data availability

All data generated or analyzed during this study are included in this published article. Images of the original Western blots used in this study are available in the Supplementary data; antibody detection characteristics across the full molecular range are available for membranes which were not pre-cut prior to immunodetection.

## Electronic supplementary material

Supplementary Western blot Images
