# Supplementary material for: Acute Physiology and Neurologic Outcomes after Brain Injury in SCOP/PHLPP1 KO Mice
Source: Sci Rep. 2018 May 8;8:7158. doi: 10.1038/s41598-018-25371-2 (PMC5940799; doi:10.1038/s41598-018-25371-2)
Supplement: Supplementary file 1 — Supplementary Western blot Images [file 41598_2018_25371_MOESM1_ESM.pdf]

**Title:** Acute Physiology and Neurologic Outcomes after Brain Injury in SCOP/PHLPP1 KO Mice

**Running Title:** Role of PHLPP1 in TBI

**Table of Contents Title:** SCOP/PHLPP1 KO Improves Memory Function after a TBI.

**Authors:** Travis C. Jackson[1,2*], C. Edward Dixon[1,3], Keri Janesko-Feldman[1,2], Vincent Vagni[1,2], Shawn E. Kotermanski[4], Edwin K. Jackson[4], Patrick M. Kochanek[1,2].

**Primary Laboratory**[1]: University of Pittsburgh School of Medicine
Safar Center for Resuscitation Research
Children's Hospital of Pittsburgh of UPMC
John G. Rangos Research Center – 6th Floor
4401 Penn Avenue
Pittsburgh, PA 15224

Secondary Location[2] University of Pittsburgh School of Medicine
Department of Critical Care Medicine
Scaife Hall
3550 Terrace Street

Secondary Location[3]: University of Pittsburgh School of Medicine
Department of Neurology
811 Kaufmann Medical Building
3471 Fifth Avenue

Secondary Location[4]: University of Pittsburgh School of Medicine
Department of Pharmacology and Chemical Biology
Bridgeside Point Building 1
100 Technology Drive

**Corresponding Author:** Travis C. Jackson, PhD*****
Assistant Professor
University of Pittsburgh School of Medicine
Safar Center for Resuscitation Research
Children's Hospital of Pittsburgh of UPMC
John G. Rangos Research Center – 6th Floor
4401 Penn Avenue
Pittsburgh, PA 15224
Email: jacksontc@upmc.edu

FIGURE 2: Western Blots

**Fig. 2A (SCOP)**

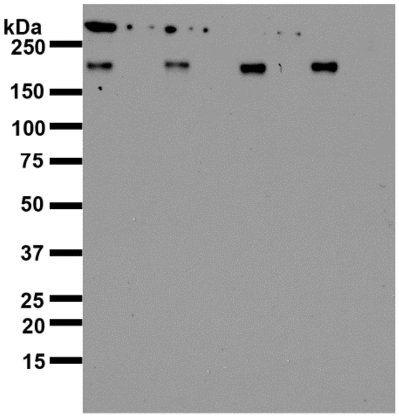


- SCOP
- Blot 1
- Probe 1

**Fig. 2A (Tubulin)**

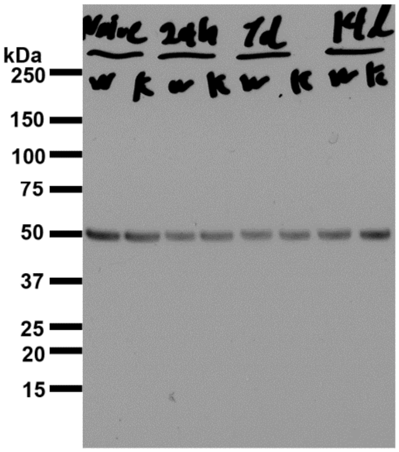


- Tubulin
- Blot 1
- Probe 2

FIGURE 4: Western Blots

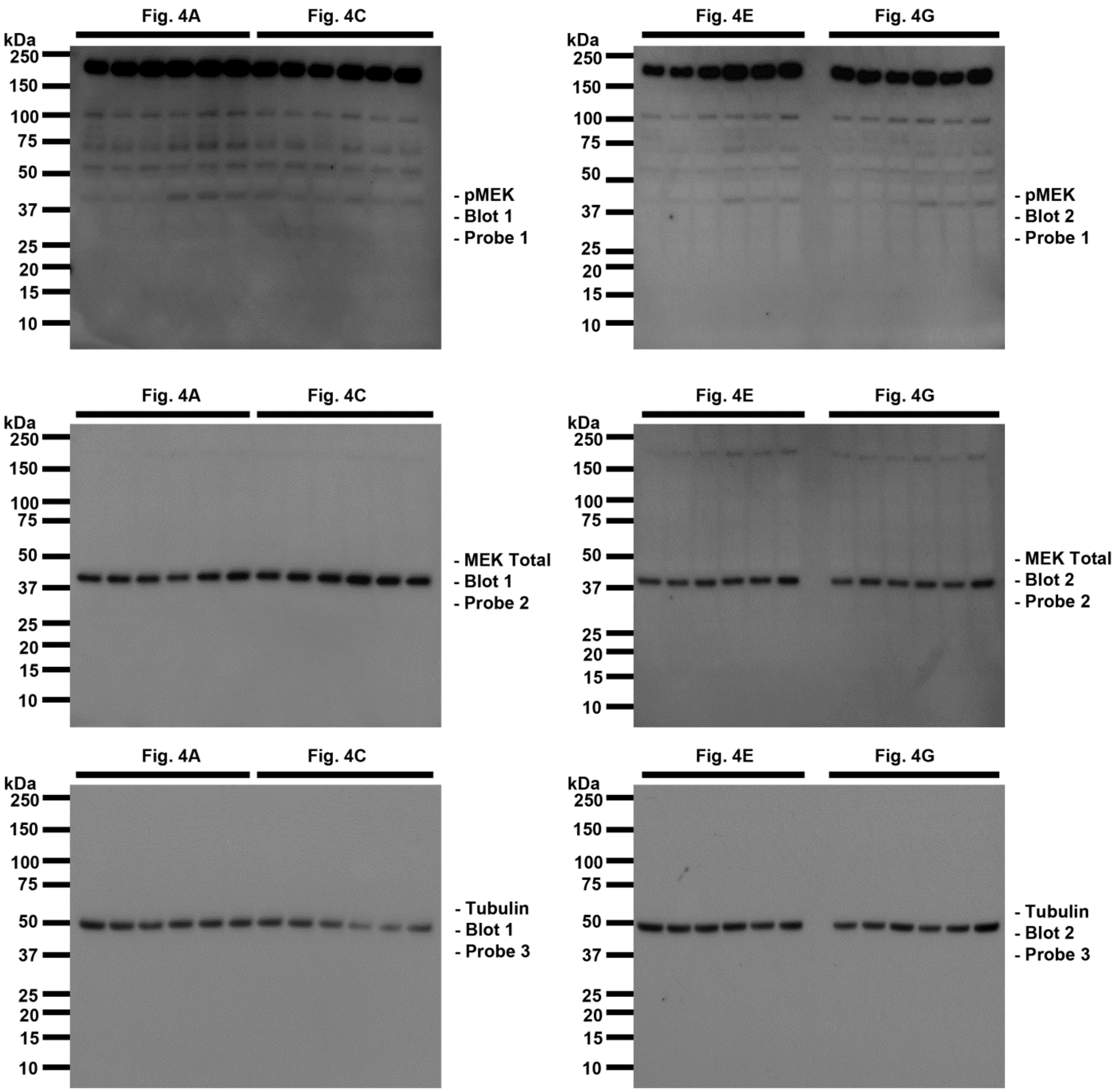

Figure 6: Sectioned PVDF Membranes (i.e. Not Cropped in Photoshop)

SCOP Levels in 7d & 14d Post-injury WT vs. KO Samples

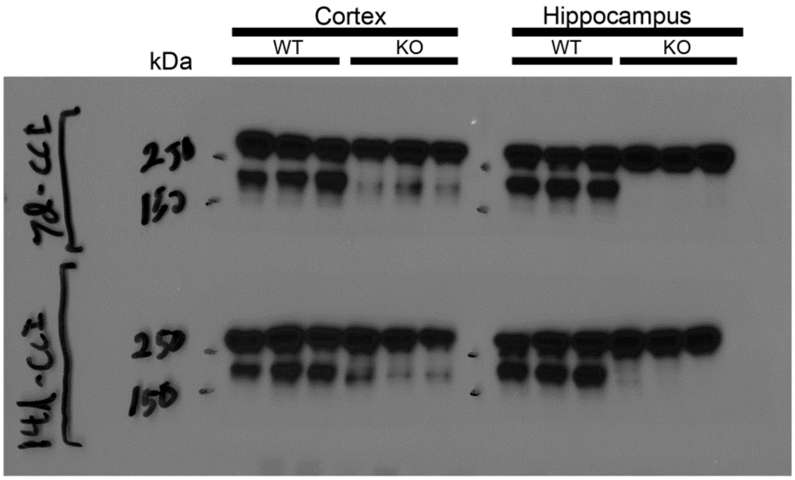


- SCOP
- Blot 1
- Probe 2

- SCOP
- Blot 2
- Probe 2

30s Exposure

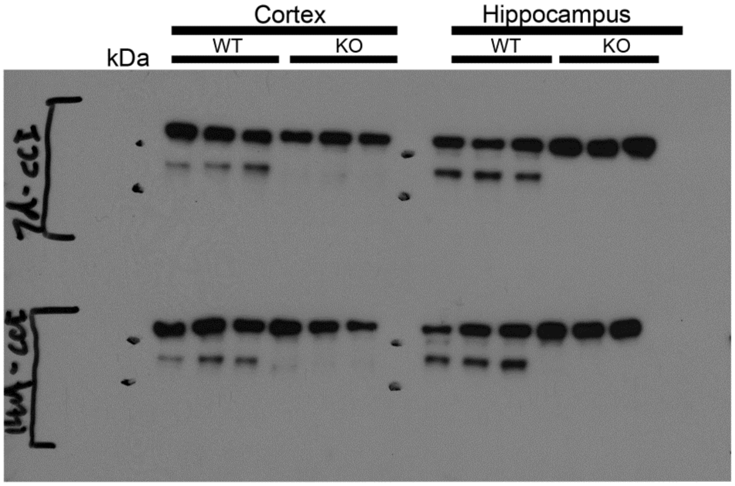


- SCOP
- Blot 1
- Proble 2

- SCOP
- Blot 2
- Probe 2

1s Exposure
(Used in Fig. 6)

Figure 6: Sectioned PVDF Membranes (i.e. Not Cropped in Photoshop)

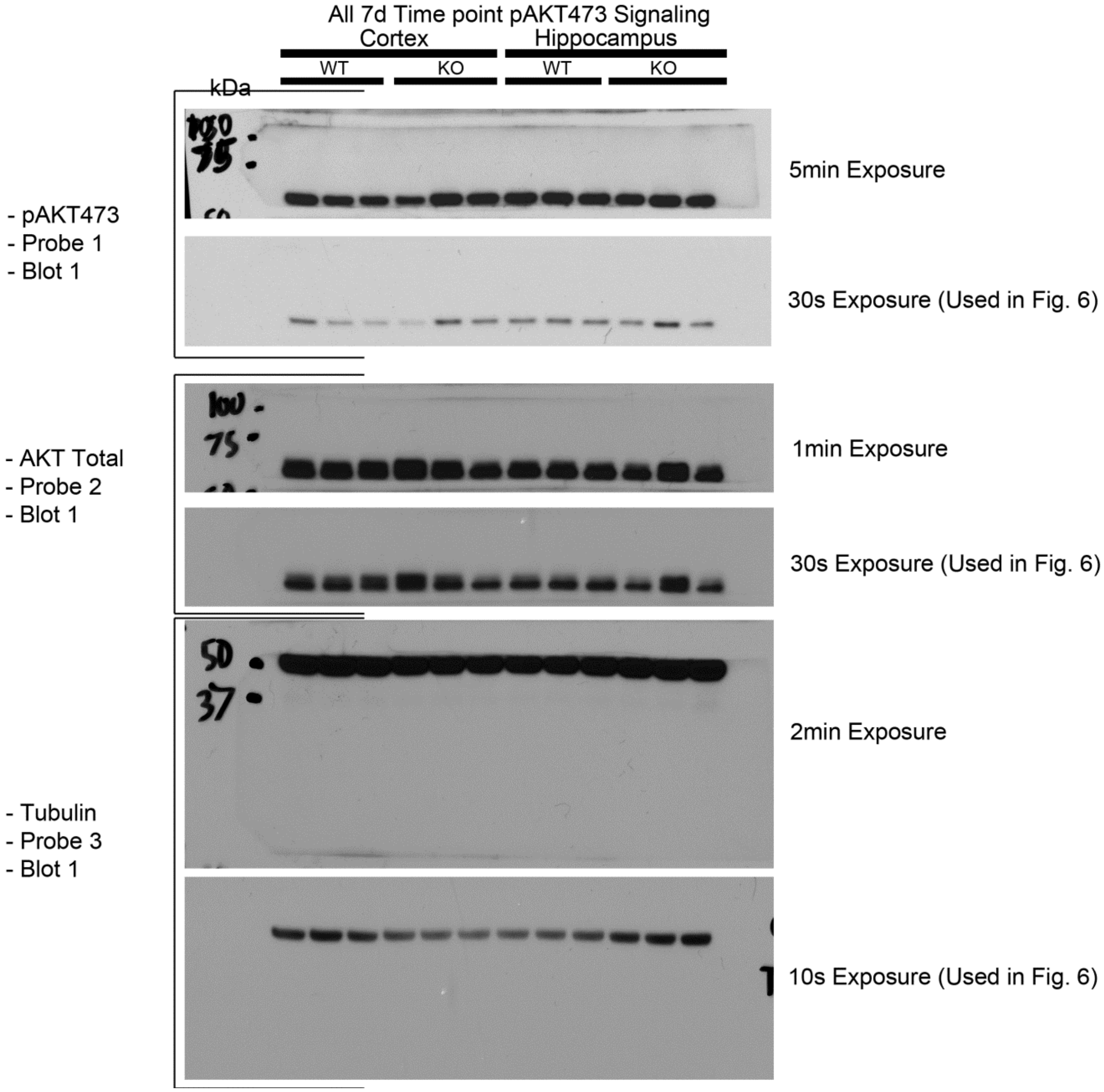

Figure 6: Sectioned PVDF Membranes (i.e. Not Cropped in Photoshop)

All 14d Time point pAKT473 Signaling

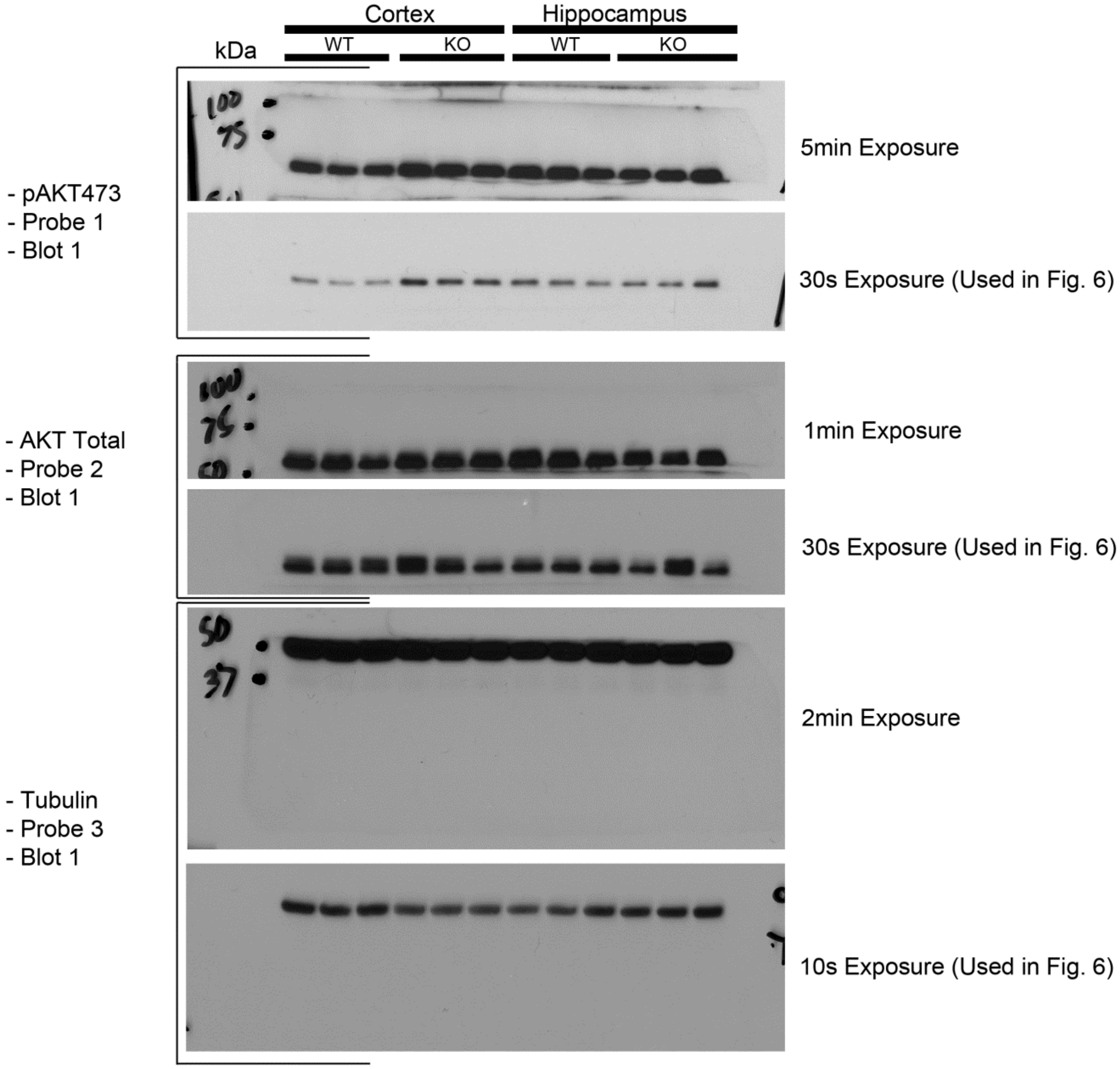

Figure 6 pAKT308 Signaling

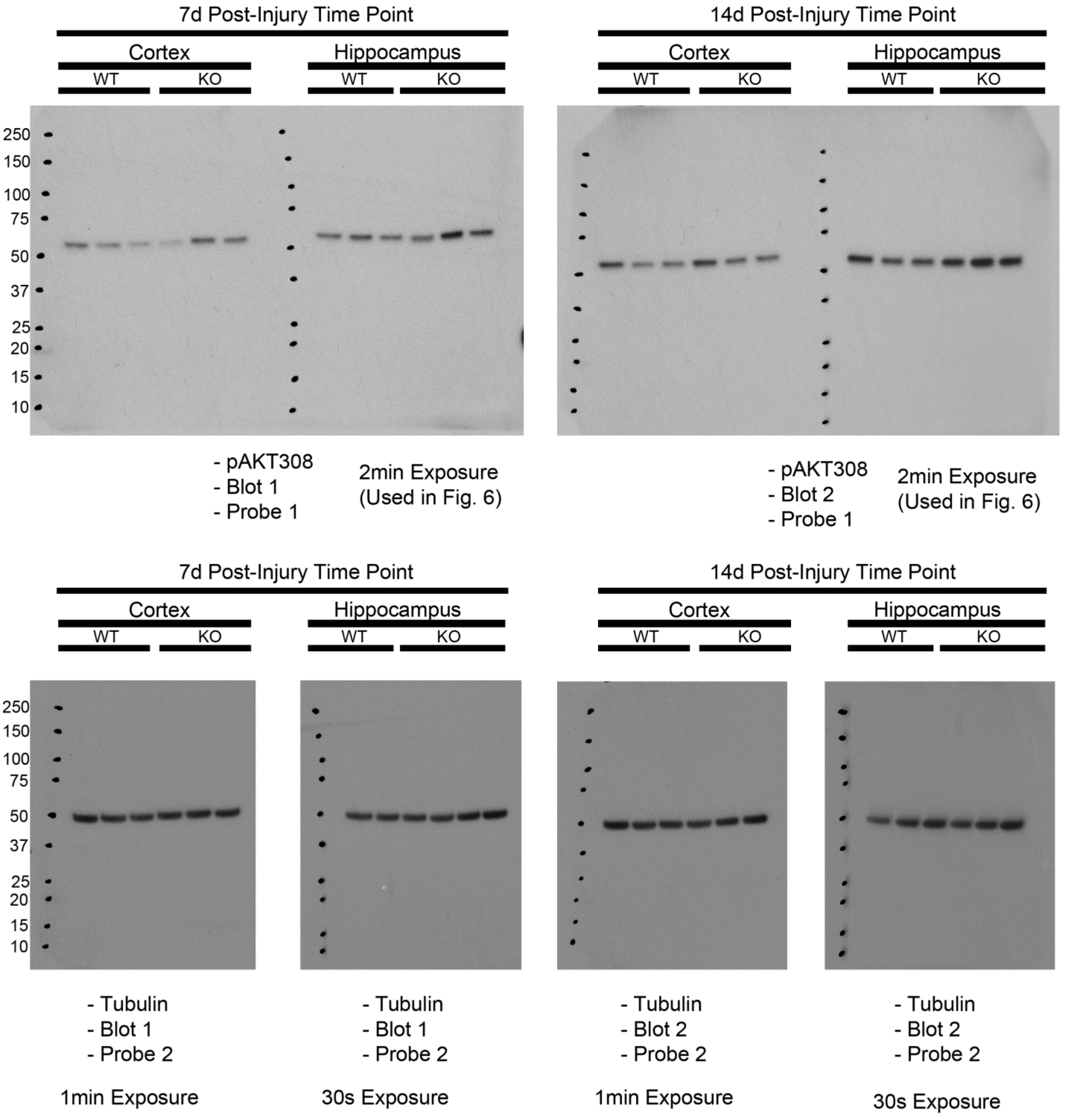

Figure 9: Western blot

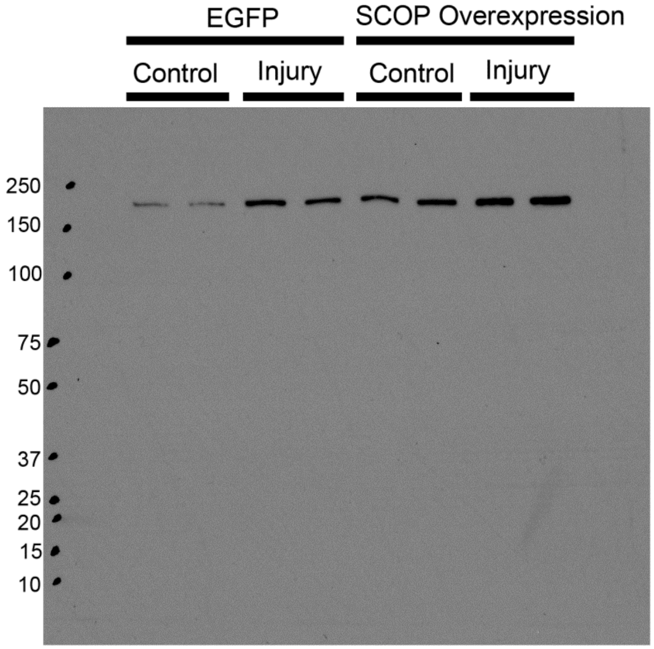


- SCOP
- Blot 1
- Probe 1

2.5min Exposure (Used in Fig. 9)
